# Influence of Specific Collagen Peptides and Concurrent Training on Cardiometabolic Parameters and Performance Indices in Women: A Randomized Controlled Trial

**DOI:** 10.3389/fnut.2020.580918

**Published:** 2020-11-19

**Authors:** Patrick Jendricke, Jan Kohl, Christoph Centner, Albert Gollhofer, Daniel König

**Affiliations:** ^1^Department of Sports and Sport Science, University of Freiburg, Freiburg, Germany; ^2^Department of Sports Science, Institute for Nutrition and Sports, University of Vienna, Vienna, Austria

**Keywords:** running distance, endurance performance, lactate threshold, body composition, concurrent training, collagen peptide, protein supplementation

## Abstract

The purpose was to examine the effects of concurrent training (CT) combined with specific collagen peptides (SCP) intake on cardiometabolic parameters and performance indices in women. In a double-blind, placebo-controlled, randomized trial recreationally active women (*n* = 59) completed a 12-week CT training (3 day/week) and ingested 15 g of SCP (treatment group [TG]) or placebo (control group [CG]) on a daily basis. Running distance as a marker of endurance performance (time trial), velocity and heart rate at the lactate and anaerobic threshold (incremental running test) and body composition (bioelectrical impedance analysis [BIA]) were measured. BIA measurements included determination of fat mass (FM) and fat free mass (FFM). Additionally, muscular strength (one-repetition-maximum [1RM]) and muscular endurance (60% of 1RM) were assessed. After 12-weeks, TG had a higher increase in running distance (1,034 ± 643 m) compared to the CG (703 ± 356 m) indicated by a significant interaction effect (*p* < 0.05). Velocity at lactate and anaerobic threshold improved in both groups over time (*p* < 0.001), with no significant differences between groups. Similarly, heart rate at lactate threshold decreased over time (*p* < 0.001), with no time × group interaction. TG declined more in heart rate at anaerobic threshold (−8 ± 14 bpm) than the CG (−1 ± 7 bpm), which resulted in a significant interaction effect (*p* < 0.01). FM decreased over time in TG and CG (*p* < 0.001), with no group differences. On contrary, TG had a higher increase in FFM (0.8 ± 0.9 kg) compared to the CG (0.3 ± 1.0 kg) (time × group interaction: *p* < 0.05). Both, 1RM and muscular endurance improved over time (*p* < 0.001), with no significant group differences. In conclusion, supplementation of SCP in combination with CT resulted in a significant increase in endurance performance compared to the control group. This might potentially be a consequence of improved structural and cardiometabolic adaptations.

## Introduction

Since 1975, obesity has nearly tripled worldwide according to World Health Organization (WHO). Physical inactivity and poor nutritional practices are considered to be major contributing factors (1). Besides lifestyle-related factors, the physiological aging process leads to an unfavorable change in body composition. Scientific evidence indicates that the age-related loss in muscle mass and function (sarcopenia) (2) and the increase in fat mass cause a majority of chronic degenerative diseases. Obesity exacerbates sarcopenia and enhances the infiltration of fat into the muscle (3).

According to the WHO global recommendations on physical activity for health in adults, resistance and endurance exercises seem to be an effective strategy to improve metabolic health (4). Typically, resistance training induces hypertrophic adaptations (5) that augment total energy expenditure, as fat free mass is a main determinant of resting metabolic rate (6). In contrast, endurance training increases muscle aerobic capacity and mitochondrial content (7) that shift fuel utilization toward fat metabolism at rest and during submaximal exercise (8–11). Comparisons between running and cycling have shown a higher rate of fat oxidation during running at the same relative intensity, which is associated with higher total energy expenditure (10). Especially in premenopausal women, these effects seem to occur through direct adaptions in the lipid metabolic pathway or indirectly through a reduction in fat mass. Investigating the underlying mechanisms of these metabolic adaptations, it is generally accepted that the use of bigger muscle groups during the eccentric muscle loading phase in running causes larger increases in the fat oxidation, whereas in cycling concentric muscle loading phase are in the foreground (12, 13). In addition, this divergence in contraction types in running compared to cycling may also provide a sufficient mechanical stimulus for collagen synthesis in the connective tissue (14).

Recent research suggests that protein intake may have synergistic effects on the maintenance and promotion of muscle growth, reducing a catabolic metabolic state and optimize the regeneration in the post-exercise phase following resistance and endurance training (15–17). Given the small body of research currently available, further long-term investigations are needed which strengthen the evidence of protein supplementation on physiological outcomes and performance parameters. Regarding the type of protein, whey, soy and casein have been investigated (18–20). Recently, collagen peptides have also received attention due to their beneficial influence on body composition during resistance training regimes (21, 22) and recovery of muscle function following strenuous physical exercise (23). However, the exact physiological mechanisms how collagen peptides affect body composition and muscular performance in humans have not been fully elucidated. In particular, the fact that collagen peptides are not a complete protein and the small concentration of some amino acids, especially leucine, has been cited as a point of criticism.

Collagen peptides are produced for nutritional administration by a specific hydrolysis of collagen. Therefore, due to the small molecule size and lack of cross-links, collagen peptides have a high biological availability, which has been demonstrated in preclinical (24, 25) and clinical studies (26, 27). In addition, collagen di- and tripeptides enter the bloodstream via the peptide transporter 1 or in the form of free amino acids (28–30). From a molecular point of view, collagen peptides are high in glycine, proline and hydroxyproline (31). Both, hydroxyproline and glycine have been measured in human blood in considerable amounts following ingestion of collagen peptides (32). Collagen-derived peptides have shown to accumulate in protein and collagen-containing tissues such as skin, cartilage or muscle (30, 33). Moreover, first clinical interventions have demonstrated, that the ingestion of specific collagen peptides has induced an upregulation in the synthesis of proteins, which are predominantly associated with the metabolism of contractile elements (22). Previously, an *in vitro* investigation suggested that the collagen derived peptide hydroxyprolyl-glycine seems to stimulate mTOR phosphorylation in myoblasts (34).

Most recently, an *in vivo* study demonstrated a significant increase in fat free mass and strength as well as an above average decrease in fat mass when supplementing specific collagen peptides (SCP) during a 12-week resistance training period (21). With respect to a significant decrease in fat mass, a direct influence on fat metabolism by SCP could be presumed as a possible cause. It has been shown that glycine, an amino acid particularly high in collagen peptides, stimulates the secretion of glucagon by increasing glucagon-like peptide-1 (GLP-1) (35). It is well established that signals such as GLP-1 activates transcription factors and increase peroxisome proliferator co-activator-1α (PGC-1α) expressions (7, 36). In addition, the interaction of PGC-1α with peroxisome proliferator-activated receptor (PPAR) stimulates fatty acid utilization which is accompanied by an increased fat metabolism (37), especially after ingestion of SCP (38, 39).

Therefore, despite the fact of e.g. low leucine concentration, this poses the question whether the supplementation of SCP might also influence cardiometabolic parameters and performance indices in endurance runners. As a previous meta-analysis has demonstrated that plain endurance training is often associated with smaller gains in lower body strength and hypertrophy than strength and concurrent training alone (40), we included resistance training features in the exercise program in order to simultaneously maintain or even improve both parameters. Long-term trials from our and other laboratories have revealed that SCP enhances strength outcomes when combined with resistance exercise (21, 22). Thus, we hypothesize that a concurrent training (CT) with endurance and resistance exercise features enhances cardiometabolic parameters, body composition and muscle strength in women when combined with a SCP supplementation. Based on these findings, the main purpose of this study was to examine the influence of CT with SCP supplementation on cardiometabolic parameters in women compared to a CT group without SCP. Changes in cardiometabolic parameters were analyzed indirectly by running distance (1), heart rate and velocity at lactate thresholds (2). Another aim was to test alterations in fat mass (3), fat free mass (4), strength (5) and muscular endurance for lower limbs (6).

## Materials and Methods

### Participants

Upon approval from the ethical committee of the University of Freiburg (ETK: 43/18-190309) 90 healthy women aged between 18 and 40 years with a BMI between 18.5 and 26 kg m^−2^ were recruited. Prior to study participation, participants read and signed informed consent. This trial was registered at the German Clinical Trials Registry (DRKS-ID: DRKS00020534).

In this investigation, exclusively recreational active female runners with a body fat percentage of > 20% were included, who regularly completed a running training twice a week for 60 min each with a minimum of running experience of 6 months. This was based on the fact that we wanted to avoid overload damage and high dropout rates. To ensure that the female runners would be amenable to training related adaptations, running activity was restricted to <120 min per week. In general, physical activity were assessed with a validated questionnaire (41). All the participants completed a comprehensive medical examination and routine blood testing for analysis of safety variables such as hemogram, creatinine, urea, aspartate transaminase, alanine aminotransferase and D-dimer.

Female runners were not eligible if they had any contraindications with regard to physical activity according to the American College of Sports Medicine (ACSM) guidelines such as cardiovascular, metabolic or renal diseases (42). Furthermore, participants were excluded from the investigation, if they had supplemented collagen peptides or other protein supplements in the past 6 months, and had intolerance or allergy against collagen peptides and silicea. Participants were also excluded from the trial if any health problems during or after physical activity or unstable weight and eating behaviors were present.

**Figure 1:**
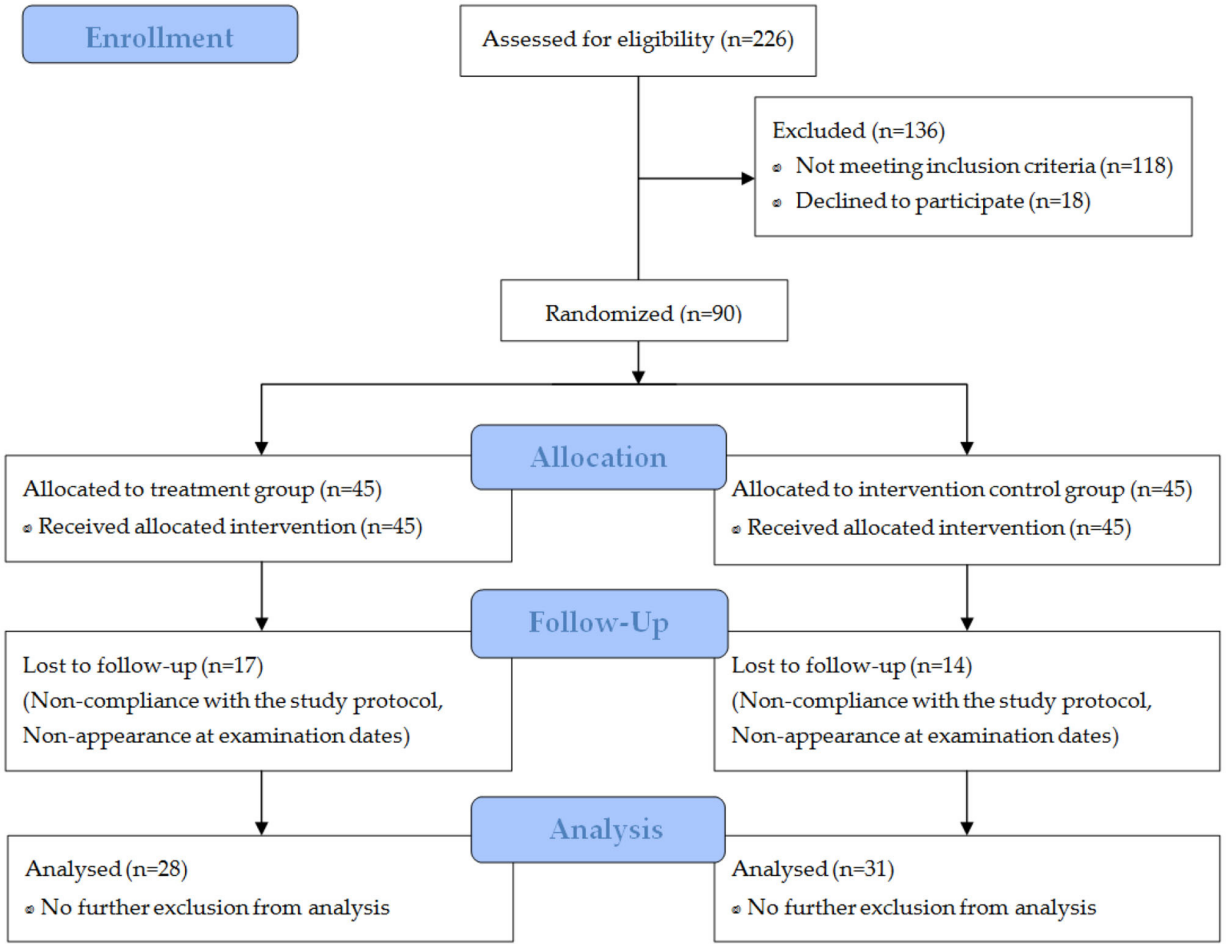
Flow chart of subject recruitment, randomization, and follow-up.

In total, 226 women wanted to take part in the study (Figure 1). After a telephone interview (prescreening), 136 subjects were not eligible. Thus, 90 participants were randomly assigned to the study groups. Due to non-compliance with the study protocol or non-appearance at the final examination date, 31 participants were excluded from the per protocol analysis.

### Study Design

The present investigation is a monocentric, prospective, placebo-controlled, double-blind, randomized trial with pre- and post-test measurements conducted at the University of Freiburg in Germany. For this purpose, a block randomization was performed using a random number generator (43). Investigators and participants were blinded until all the data had been entered and the statistical analysis had been performed.

After a preliminary screening to check the inclusion criteria, subjects were randomly assigned either into the treatment group (TG) receiving specific collagen peptides or the control group (CG) receiving silicea as placebo. Subsequently, all subjects were familiarized with testing procedures and exercise techniques by a certified sport scientist. All familiarizations were conducted in individual sessions for each test by three trials on non-consecutive days before the measurement for the study was started.

At baseline and after 12 weeks, time trial, incremental running test on a treadmill, body composition and strength testing were performed. The main outcome of this trial was the difference in time trial performance by measuring the running distance before and after the CT between the TG and the CG. As a secondary outcome, changes in heart rate and velocity at the aerobic and anaerobic threshold were measured by incremental treadmill test. In addition, the alteration in fat free mass (FFM) and fat mass was compared between the two groups. Body composition was quantified by using a bioelectric impedance analysis (BIA). Finally, one-repetition maximum testing (1RM for back squat) and muscular endurance testing (60% of 1RM for back squat) of the legs were analyzed before and after 12 weeks.

### Experimental Protocol

On three non-consecutive days (e.g., Monday, Wednesday, and Friday), time trial, incremental running test and strengths measurements were conducted in a standardized order. Each test was done in the morning after a standardized 12 h overnight fast. Furthermore, 2 days before the measurements, subjects documented their diet and exercise patterns to standardize macronutrients intake and physical activity. A standardized test meal was provided 2 h before the test. Within 10 min the test meal and water had to been consumed (44, 45).

### Time Trial

After the standardized test meal, subjects completed a 1-h time trial on a 400 m center track. Female runners were not provided visual or verbal feedback on performance times, heart rate and covered distance (46–48). Every 10 min they were asked for their rating of perceived exertion (RPE) on a 6–20 scoring Borg scale (49). Besides the evaluator, the covered distance and HR were continuously tracked by GPS (Polar M200, Kempele, Finland). All measurements were conducted in the morning with the aim of maintaining similar experimental conditions throughout. Temperatures ranging from 17–25°C and humidity were between 50 and 60%.

### Incremental Running Test

At the beginning, subjects were instructed to determine their RPE during the performance tests. Additionally, heart rate was monitored throughout all tests using a heart rate monitor (Polar M 200®, Kempele, Finland). The incremental test on the treadmill (hp cosmos quasar®, Nussdorf-Traunstein, Germany) was used to determine the velocity at lactate threshold (V_LT_), velocity at individual anaerobic threshold (V_IAT_), heart rate at lactate threshold (HR_LT_), heart rate at individual anaerobic threshold (HR_IAT_), lactate threshold (LT) and individual anaerobic threshold (IAT). Starting with 6 km·h^−1^ the velocity was increased by 2 km·h^−1^ every 3 min until exhaustion. Blood lactate concentrations were analyzed in between stages from capillary blood and were taken from the hyperemized earlobe (50). In addition, HR and RPE were continuous recorded. Incremental running tests were conducted in an air-conditioned laboratory with temperature set at 20°C and relative humidity of 50%.

### Blood Lactate Analysis

Slope of the blood lactate rise in response to exercise was used to indirectly analyze aerobic and anaerobic metabolic adaptations (51, 52). With an end-to-end plastic capillary 25 μL of capillary blood was drawn from the earlobe of each subject. Subsequently, the samples were collected in microtubes (Eppendorf Tubes®, Eppendorf, Germany) containing 50 μL of 1% sodium fluoride (NaF) for analysis of blood lactate concentration, using the enzymatic-amperometric methods via blood lactate analyzer (Biosen S-line, EKF Diagnostics, Barleben, Germany). Blood lactate concentrations were analyzed in between stages, on lactate threshold and individual anaerobic threshold. In addition, velocity and heart rate on each stage were detected. All parameters were automatically evaluated by computer software (Ergonizer 4.7.4, Freiburg, Germany). Thereby, LT reflects the first measurable increase in blood lactate concentration during physical activity (53, 54). The IAT detects the highest aerobic exercise intensity that can be maintained over time without metabolic energy from continuing net lactate production. In the present study, the IAT is determined as the velocity at a net increase in lactate concentration 1.5 mmol·l^−1^ above the lactate concentration at LT (55).

### Body Composition

Body composition measurements were performed using a procedure, which has previously been described in detail (21). Briefly, according to the guidelines of the European Society for Clinical Nutrition and Metabolism (ESPEN) (56), fat mass (FM) and fat free mass (FFM), skeletal muscle mass (SMM), total body water (TBW), extracellular water (ECW) and intracellular water (ICW) were pre- and post-tested on preliminary screening day using a seca Medical Body Composition Analyzer 515 (seca© mBCA 515, Hamburg, Germany). Standardized BIA assessments were performed after a 12-h overnight fast and each measurements were performed in the morning for the same time of day. All requirements for an accurate BIA utilization in clinical practice were stringently followed in order to reduce biological and technical error in this study (56, 57). Therefore, participants were instructed to abstain from exercising (48 h), consuming alcohol (48 h) and caffeine (12 h). Metal and accessories were removed and subjects wore tight fitting clothing. In addition, each individual was asked to void its bladder prior to testing. Before body composition analyses were conducted in a standing position with bare feet placed properly on the contact electrodes, body height was obtained with the Stadiometer (seca© 274, Hamburg, Germany) to the nearest 1 mm.

### Strength Testing

On the basis of the strength test protocol by Rana et al. (58) each subject was pre- and post-tested in back squats for 1RM and muscular endurance. The procedures utilized for each subject and test, were identical.

In preparation for the measurements, subjects completed a standardized 5-min submaximal warm-up on a cycle ergometer. Subsequently, a specific warmup for back squats was performed using a set of 8–10 repetitions (40–60% of 1RM), a set of 2–3 repetitions (75% of 1RM), and a set of 1 repetition (90% of 1RM). Final 1RM was determined as the maximum load lifted through full range of motion and was detected in less than five 1RM attempts. Following each successful lift, the load was increased by 5–10% until the subject failed to lift the load with a proper technique through the entire range of motion. Single sets separated by a 3–5 min resting period to insure adequate recovery of these sets. Moreover, the execution speed was fixed at 1.5 s for the eccentric phase and 1.5 s for the concentric phase via metronome and verbal counting by the investigator. No isometric phase was allowed during the testing.

Following assessment of 1RM, the subjects given 10 min of rest. Afterwards, the resistance was set at 60% of the subject's 1RM and they performed as many repetitions as possible until fatigue, which was quantified by the rating of perceived exertion. The muscular endurance test was conducted in an identical manner as the 1RM test. As a result, the number of repetitions performed served as the measure for muscular endurance.

### Test Meal

Endurance performance may be influenced by the previous meal. Therefore, subjects refrained from strenuous exercise, caffeine and alcohol consumption in the 24-h period before each experimental trial. In addition, subjects were restricted from food or fluid intake 12 h prior testing (45).

Two hours before the onset of each measurement subjects consumed a standardized test meal in the laboratory. The test meal contained 1 g carbohydrate per kg body weight and was similar in macronutrient content. It consists of wholegrain oats and semi skimmed milk. To ensure that each subject consumed a standardized volume of 650 ml fluid within 10 min, a specific amount of water and milk with each test meal was prepared (44).

### Test Product Supplementation

The test product used in this study (SCP; Gelita AG, Eberbach, Germany) is composed of specific collagen peptides with a high safety (GRAS status). During the 12-weeks intervention subjects daily supplemented either 15 g of SCP or 15 g of silicea as a placebo (silicon dioxide). Two hours before each training session, both groups ingested 7.5 g dissolved in 250 ml water. Immediately after each exercise session another 7.5 g were consumed. Similarly, on days without training subjects were also instructed to supplement doses in a split fashion at the same time as on training days. The timing of the pre and post exercise beverages were corresponded to the recommendation of Iwai et al. (26), with the aim of ensuring a high amount of collagen peptides in human blood before and after each training session. On days without training, subjects were instructed to consume the drinks at the same time as on the day before. All of the subjects were blinded to which supplementation was ingested during the investigation. Supplementations had similar color and taste.

### Dietary Intake and Energy Expenditure

Before and after the intervention subjects recorded their dietary intake for two weekdays and one weekend day on three consecutive days to gain perspective on eating habits. In doing so, all subjects were given detailed instructions from a nutritionist on how to quantify food proportions and thoroughness of meal description needed for precise computation. Subjects were instructed not to add the test product supplementation to their food logs. Energy and macronutrient intakes were examined using Nutriguide 4.6 (Nutri Science GmbH, Hausach, Germany).On the basis of this information, total energy intake, protein, amino acids, carbohydrate, fat and fluid intake were calculated.

Besides dietary intake, subjects documented their physical activity before and after the investigation to gain perspective on physical activity habits. Physical activity was measured by the self-reported Freiburg Questionnaire of Physical Activity (FFKA) (41). The FFKA contained 12 standardized questions, including basic, leisure, and sports-type activities. Additionally, subjects were also instructed not to add the strength and endurance training to the FFKA, which were part of the intervention. According to the Compendium of Physical Activities, we determined the energy expenditure. This Compendium provides a five-digit coding scheme linking categories and types of physical activity with their respective metabolic equivalent of task (MET) intensity values. By multiplying the bodyweight in kg by the MET intensities values and duration of physical activity, we estimated the kcal of energy expenditure to the subject's individual bodyweight per day (59, 60).

Based on the data of the pre-examination, subjects were encouraged to maintain their dietary and physical activity habits during the study.

### Training Protocol

Over a period of 12 weeks, subjects performed a supervised concurrent resistance and endurance training, three times a week with at least 1 day of rest between two sessions.

Each training session started with the resistance exercises based on Klika and Jordan (61), performing 3 sets squats, lunges and one legged heel rises using the subject's bodyweight as resistance. During the training period, number of repetitions gradually increased: week 1–4: 20 repetitions, week 5–8: 25 repetitions, and week 9–12: 30 repetitions. Loading strategies, with respect to sets and repetition range, were in accordance with previous recommendations for increasing muscular endurance (5, 62). Subjects were encouraged to maintain an execution speed of 2 s, which was equally split between concentric and eccentric phase. Between the sets a 30 s rest interval was provided.

After the resistance training 1 h endurance training was performed on a 400 m running track. Individual anaerobic threshold was determined during the training period, intensities progressively increased from week 1–4: 80% of VIAT, week 5–8: 85% of VIAT and week 9–12: 90% of VIAT (63, 64).

All training sessions were conducted at the University of Freiburg and were supervised by highly experienced exercise instructors.

### Statistical Analysis

Per protocol analyses were utilized using IBM SPSS Statistics 25 (IBM, Armonk, NY, USA). Data are presented as mean ± standard deviation (SD) in tables and figures. The level of significance was set to α < 0.05 for all performed two-sided tests. Since the variable data of all groups showed normal distribution according to the results of Kolmogorov-Smirnoff test, the homogeneity of the baseline values between study groups was checked via independent *t*-tests. To detect changes over time and respective differences between the groups, a repeated-measures ANOVA (rmANOVA) with factors time (pre, post) × group (TG, CG) was performed to test for interaction effects. In the case of significant interaction effects from the rmANOVA, Bonferroni corrected Student's *t*-tests were calculated for any pre to post differences. In order to describe, if effects have a relevant magnitude, Cohen's d was conducted between the groups. If both groups improved significantly, Cohen's d was performed within the groups.

## Results

### Subject Characteristics

In total, 59 subjects completed the investigation and were included in the per protocol analysis. Neither the TG (*n* = 28) nor the CG (*n* = 31) differed statistically significantly in age, height, weight, body mass index (BMI), FM, FFM or hydration status (Table 1). Furthermore, baseline data of the outcomes did not significantly differ between the groups except for the HR at IAT (**Table 3**). All 31 dropouts failed to comply with the study design, mainly missing too many (<80%) training days or could not perform their training protocol sufficiently due to illness, injury or other reasons. No dropout was related to side effects of the supplemented SCP or placebo and no pathological findings were observed in the routine blood test.

**Table 1:** Baseline characteristics of the subjects in TG (*n* = 28) and CG (*n* = 31).

**Parameter**	**Group**	**(M ± SD)**	**MIN**	**MAX**
Age (years)	TG	25.4 ± 4.2	18	35
	CG	26.8 ± 5.7	20	39
Height (cm)	TG	167 ± 6.8	153	180
	CG	167 ± 6.3	156	181
Body weight (kg)	TG	62.5 ± 8.6	46.4	78.5
	CG	63.3 ± 6.0	52.5	74.4
BMI (kg·m^−2^)	TG	22.2 ± 2.1	18.7	26.0
	CG	22.6 ± 1.6	18.7	24.9
FM (kg)	TG	17.9 ± 5.19	10.1	29.9
	CG	17.7 ± 3.79	10.8	26.5
FFM (kg)	TG	44.6 ± 4.69	36.2	56.9
	CG	45.6 ± 4.33	37.1	54.5

Values are means ± SD for n subjects. TG, treatment group; CG, control group; BMI, body mass index; FM, fat mass; FFM, fat free mass.

### Dietary Intake and Energy Expenditure

As illustrated in Table 2, the groups did not show significant differences in energy intake, macronutrients, amino acids, fluid intake and energy expenditure at baseline (*p* > 0.05). After the intervention, no significant main effect of time (*p* > 0.05) or time × group interaction (*p* > 0.05) was determined for these lifestyle-related parameters.

**Table 2:** Changes in energy intake, macronutrients, amino acids, fluid intake, and energy expenditure before (Pre) and after (Post) supplementation with TG (*n* = 28) or CG (*n* = 31).

**Parameter**	**Group**	**Pre**	**Post**	**Main effect (Time)**	**Interaction effect (Time × Group)**	***d***
Total energy (kcal)	TG	1,849 ± 565	1,757 ± 644	0.473	0.676	0.112
	CG	1,795 ± 533	1,771 ± 484			
Protein intake (g)	TG	63.3 ± 23.7	59.8 ± 19.2	0.208	0.804	0.066
	CG	65.5 ± 23.8	60.3 ± 18.4			
Fat intake (g)	TG	70.8 ± 33.7	69.4 ± 31.9	0.959	0.750	0.085
	CG	67.6 ± 27.7	68.7 ± 23.5			
Carbohydrate intake (g)	TG	211 ± 73.4	199 ± 78.8	0.356	0.872	0.043
	CG	209 ± 65.5	201 ± 67.6			
Leucine (g)	TG	4.97 ± 1.91	4.68 ± 1.49	0.220	0.815	0.062
	CG	5.04 ± 2.03	4.62 ± 1.49			
BCAA (g)	TG	11.0 ± 4.86	10.7 ± 3.43	0.373	0.643	0.124
	CG	11.7 ± 4.68	10.8 ± 3.17			
Fluid intake (ml)	TG	2,486 ± 1,180	2,307 ± 1,357	0.772	0.067	0.495
	CG	2,441 ± 978	2,686 ± 1,026			
Energy expenditure (kcal)	TG	2,134 ± 1,421	1,921 ± 1,033	0.079	0.416	0.217
	CG	2,762 ± 1,218	2,188 ± 1,270			

*Values are means ± SD for n subjects. TG, treatment group; CG, control group; BCAA, branched chain amino acids; d, Cohen's d*.

### Time Trial

**Figure 2:**
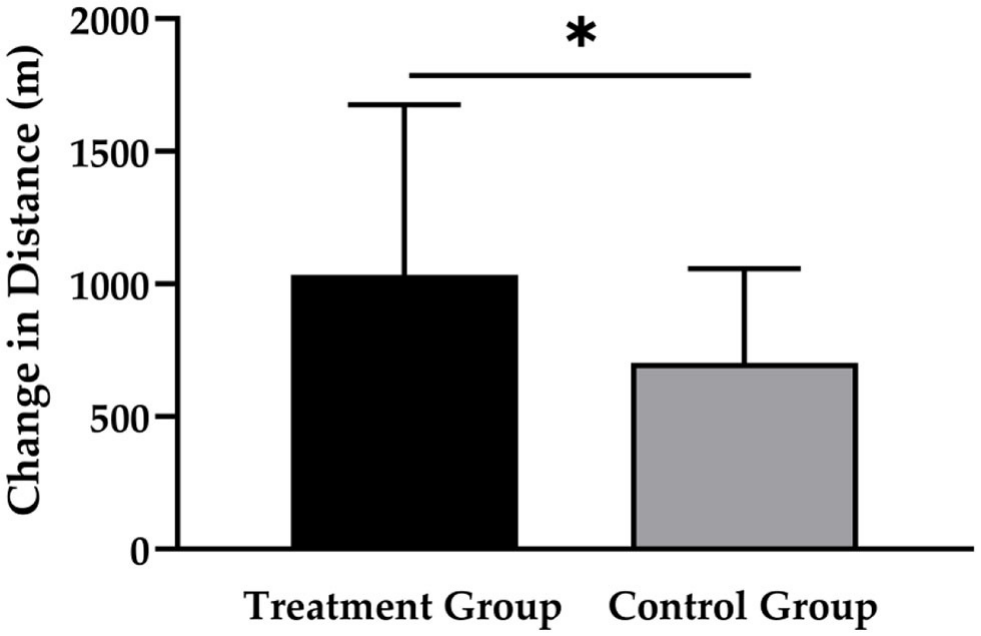
Changes in running distance after 12 weeks of intervention in treatment group (*n* = 28) and control group (*n* = 31). Values are means ± SD for n subjects. *Significantly different (*p* < 0.05) by repeated-measures ANOVA (time × group interaction).

As shown in Figure 2, the TG had a greater increase in running distance (1,034 ± 643 m; *p* < 0.001; *d* = 0.860) compared to the CG (703 ± 356 m; *p* < 0.001; *d* = 0.507), which resulted in a statistically significant main effect of time (*p* < 0.001) and time × group interaction (*p* < 0.05; *d* = 0.658).

### Incremental Running Test

An elevation in the velocity at LT was observed in both TG (0.71 ± 0.70 km·h^−1^) and CG (0.54 ± 0.60 km·h^−1^), with a significant main effect of time (*p* < 0.001), but with no significant time × group effect (*p* > 0.05; *d* = 0.198). Similarly, velocity at IAT increased by 0.86 ± 0.61 km·h^−1^ in the TG and by 0.77 ± 0.54 km·h^−1^ in the CG with a significant main effect of time (<0.001), but with no significant time × group effect (*p* > 0.05; *d* = 0.155) (Table 3).

**Table 3:** Changes in time trial, lactate thresholds and muscle strength before (Pre) and after (Post) supplementation with TG (*n* = 28) or CG (*n* = 31).

**Parameter**	**Group**	**Pre**	**Post**	**Main effect (Time)**	**Interaction effect (Time × Group)**	**d**
Time trial (m)	TG	8,572 ± 1,224	9,606 ± 927*	<0.001	0.016	0.658
	CG	8,929 ± 1,399	9,632 ± 1,354*			
V_LT_ (km·h^−1^)	TG	6.75 ± 0.76	7.46 ± 0.98	<0.001	0.459	0.198
	CG	6.91 ± 0.86	7.48 ± 0.99			
V_IAT_ (km·h^−1^)	TG	9.39 ± 1.12	10.25 ± 1.18	<0.001	0.562	0.155
	CG	9.55 ± 1.30	10.32 ± 1.19			
HR_LT_ (bpm)	TG	157 ± 14.2	149 ± 12.8	0.002	0.087	0.462
	CG	150 ± 15.5	148 ±12.7			
HR_IAT_ (bpm)	TG	179 ± 10.5	171 ± 13.9*	0.002	0.007	0.745
	CG	173 ± 11.1^#^	172 ± 8.96			
LT (mmol·l^−1^)	TG	1.82 ± 0.59	1.79 ± 0.51	0.443	0.802	0.067
	CG	1.78 ± 0.51	1.75 ± 0.66			
IAT (mmol·l^−1^)	TG	3.32 ± 0.59	3.25 ± 0.51	0.442	0.800	0.068
	CG	3.29 ± 0.51	3.25 ± 0.66			
1RM (kg)	TG	55.2 ± 11.2	60.2 ± 9.92	<0.001	0.243	0.313
	CG	54.7 ± 13.1	61.3 ± 12.9			
60% of 1RM (RP)	TG	22.3 ± 7.85	30.4 ± 8.99	<0.001	0.126	0.412
	CG	23.6 ± 9.67	28.7 ± 10.1			

Values are means ± SD for n subjects. TG, treatment group; CG, control group; V_LT_, velocity at lactate threshold; V_IAT_, velocity at individual anaerobic threshold; HR_LT_, heart rate at lactate threshold; HR_IAT_, heart rate at individual anaerobic threshold; LT, lactate threshold; IAT, individual anaerobic threshold; 1RM, one repetition maximum; 60% of 1RM (RP), repetitions of 60% of 1RM;

# significant difference at baseline between TG and CG;

* significant Bonferroni corrected Student's t-test; d, Cohen's d.

**Figure 3:**
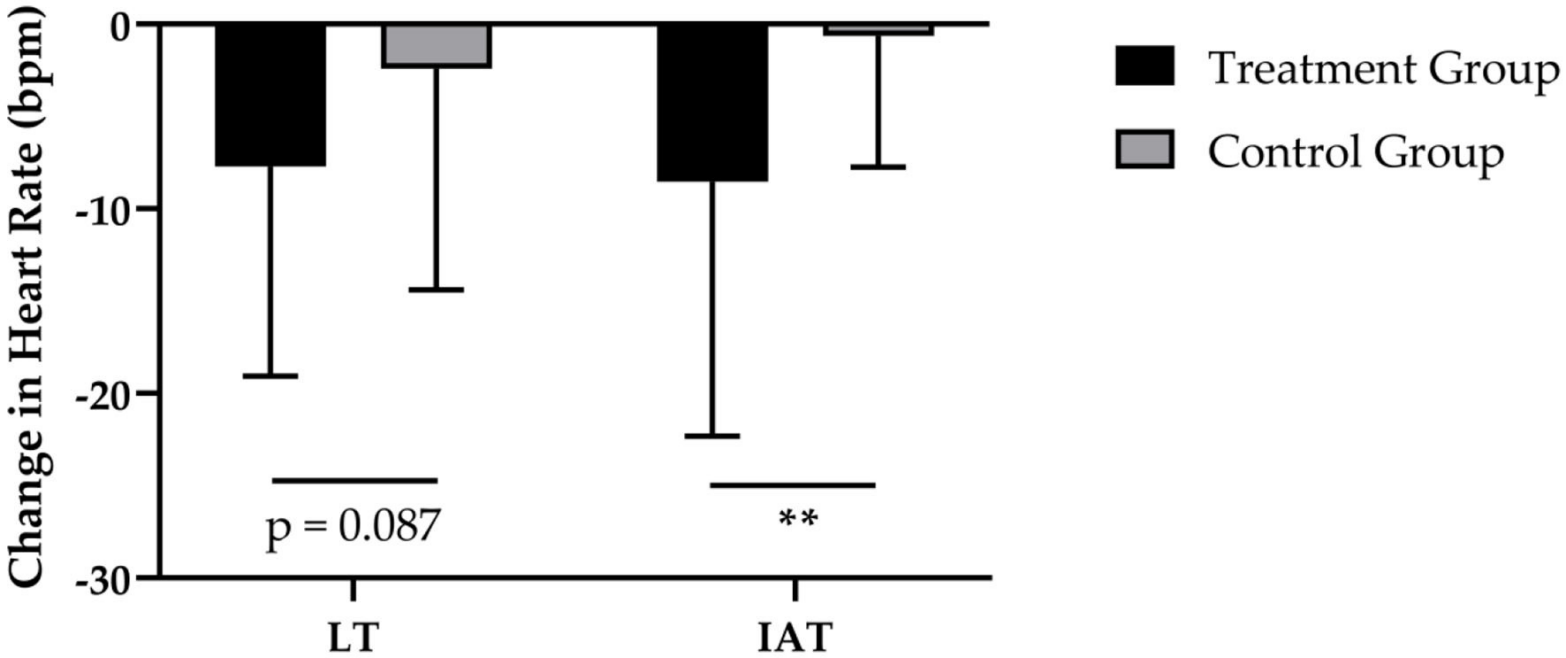
Changes in HR at LT and HR at IAT after 12 weeks of intervention in treatment group (*n* = 28) and control group (*n* = 31). Values are means ± SD for n subjects. **Significantly different (*p* < 0.01) by repeated-measures ANOVA (time × group interaction).

After 12-weeks of CT, the administration of SCP was accompanied by a reduction in HR at LT by −8 ± 11 bpm and a decrease by −2 ± 12 bpm of HR in the CG (Figure 3). These results revealed a statically relevant time effect (*p* < 0.01) with no time × group interaction (*p* > 0.05; *d* = 0.462). Furthermore, the TG had a higher decrease in HR at IAT (−8 ± 14 bpm; *p* < 0.01) in contrast to the CG (−1 ± 7 bpm; *p* > 0.05), which resulted in a significant time (*p* < 0.01) and time × group (*p* < 0.01; *d* = 0.745) effect.

A marginal decrease in LT was shown in both TG (0.03 ± 0.54 mmol·l^−1^) and CG (0.03 ± 0.59 mmol·l^−1^), with no significant time (*p* > 0.05) or time × group (*p* > 0.05; *d* = 0.067) effect. To the same extant, IAT reduced by 0.07 ± 0.54 mmol·l^−1^) in TG and by (0.04 ± 0.59 mmol·l^−1^) in the CG with neither a significant time (*p* > 0.05) nor time × group (*p* > 0.05; *d* = 0.068) effect (Table 3).

### Body Composition

**Figure 4:**
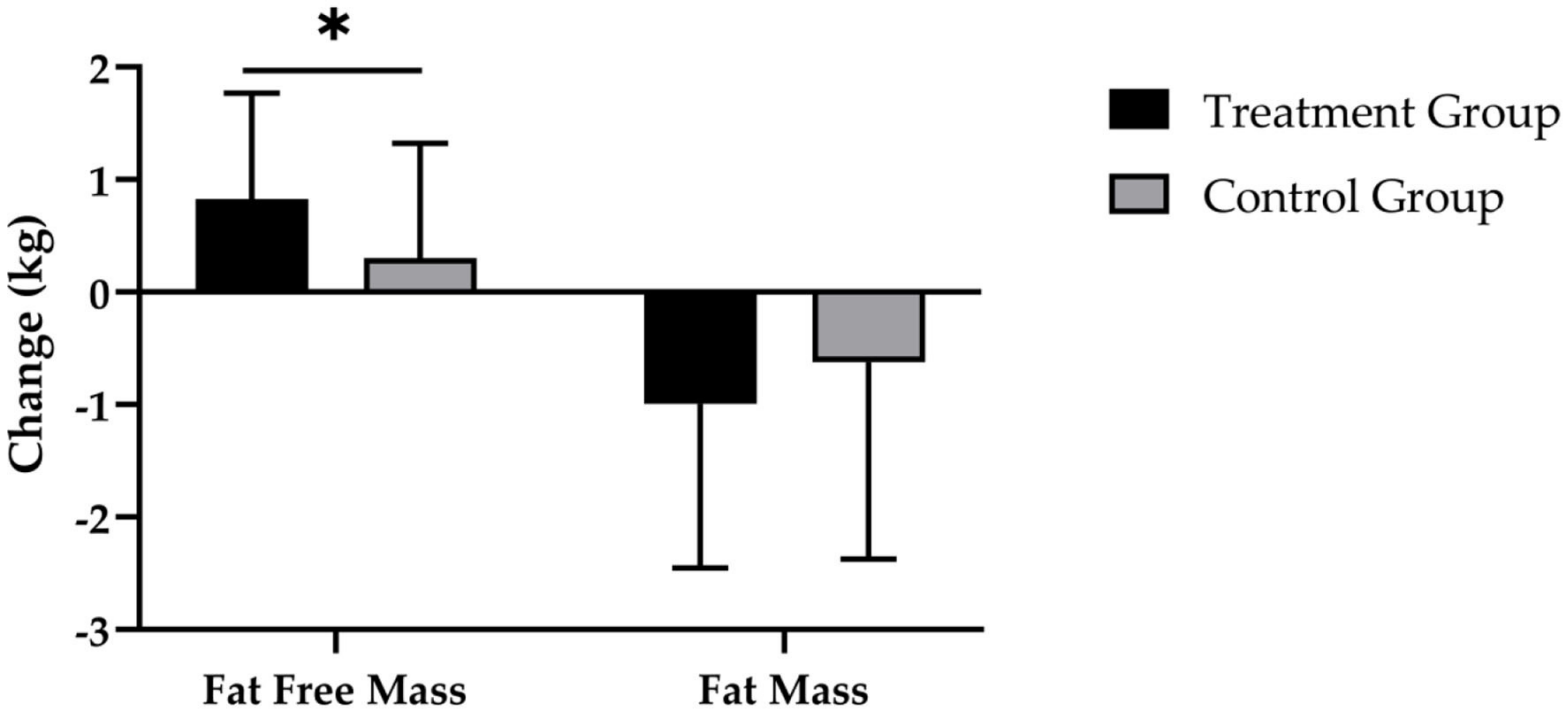
Changes in fat free mass and fat mass after 12 weeks of intervention in treatment group (*n* = 28) and control group (*n* = 31). Values are means ± SD for n subjects. *Significantly different (*p* < 0.05) by repeated-measures ANOVA (time × group interaction).

After 12 weeks of CT, the supplementation of SCP was accompanied by a reduction in FM by −1.0 ± 1.5 kg and a decrease by −0.6 ± 1.7 kg in the CG (Figure 4). Evaluation of changes in FM revealed a significant main effect of time (*p* < 0.001), with no significant time × group effect (*p* > 0.05; *d* = 0.233).

Figure 4 demonstrates that the TG had a statistically significantly higher increase in FFM (0.8 ± 0.9 kg; *p* < 0.001) compared with the CG (0.3 ± 1.0 kg; *p* > 0.05), which resulted in a statistically significant main effect of time (*p* < 0.001) and time × group interaction (*p* < 0.05; *d* = 0.543).

In addition, subjects in the TG exhibited a higher increase in SMM (0.6 ± 0.7 kg) in contrast to the CG (0.3 ± 0.7 kg). These results revealed a statistically relevant time effect (*p* < 0.001) and a strong trend toward a time × group interaction effect (p = 0.052; *d* = 0.526) (Table 4).

**Table 4:** Changes in body composition before (Pre) and after (Post) supplementation with TG (*n* = 28) or CG (*n* = 31).

**Parameter**	**Group**	**Pre**	**Post**	**Main effect (Time)**	**Interaction effect (Time × Group)**	**d**
Body weight (kg)	TG	62.5 ± 8.60	62.3 ± 8.60	0.180	0.444	0.205
	CG	63.3 ± 6.04	63.0 ± 6.63			
FM (kg)	TG	17.9 ± 5.19	16.9 ± 5.31	<0.001	0.384	0.233
	CG	17.7 ± 3.79	17.1 ± 4.13			
FFM (kg)	TG	44.6 ± 4.69	45.4 ± 4.60*	<0.001	0.045	0.543
	CG	45.6 ± 4.33	45.9 ± 4.36			
SMM (kg)	TG	21.0 ± 2.80	21.6 ± 2.85	<0.001	0.052	0.526
	CG	21.8 ± 2.58	22.1 ± 2.62			
TBW (L)	TG	32.8 ± 3.54	33.4 ± 3.51*	0.001	0.041	0.555
	CG	33.7 ± 3.30	33.9 ± 3.51			
ECW (L)	TG	14.0 ± 1.54	14.3 ± 1.65	0.001	0.383	0.234
	CG	14.3 ± 1.49	14.5 ± 1.48			
ICW (L)	TG	18.8 ± 2.03	19.1 ± 1.96*	0.101	0.011	0.694
	CG	19.4 ± 1.98	19.4 ± 2.01			

Values are means ± SD for n subjects. TG, treatment group; CG, control group; FM, fat mass; FFM, fat free mass; SMM, skeletal muscle mass; TBW, total body water; ECW, extracellular water; ICW, intracellular water; *significant Bonferroni corrected Student's t-test; d, Cohen's d.

Moreover, TBW increased by 0.6 ± 0.8 L in the TG (Table 4). In the CG a rise by 0.2 ± 0.8 L was detected. There was a significant main effect of time (*p* < 0.001) and time × group effect (*p* < 0.05; *d* = 0.555).

An increase in the ECW was observed in both the TG (0.3 ± 0.6 L) and CG (0.2 ± 0.5 L), with a significant main effect of time (p = 0.001), but with no significant time × group effect (*p* > 0.05; *d* = 0.234) (Table 4).

In contrast, the 3-month administration of SCP was accompanied by an increase of ICW by 0.3 ± 0.5 L (*p* < 0.05), while no changes were revealed in the CG (Table 4). Calculation of a repeated measures ANOVA revealed no statistically significant main effect of time (*p* > 0.05), but a significant time × group interaction (*p* < 0.05; *d* = 0.694).

### Strength Testing

As shown in Table 3, 1RM in leg strength was improved in both TG (5.0 ± 5.2) and CG (6.6 ± 6.3 kg). The results of the rmANOVA showed a significant time effect (*p* < 0.01), whereas no significant time × group effect (*p* > 0.05; *d* = 0.313) was identified.

Finally, muscle endurance in the lower limbs enhanced by 8.1 ± 7.4 rpm in the TG (Table 3). In the training group without SCP administration an increase by 5.1 ± 7.5 rpm was observed. There was a significant main effect of time (*p* < 0.01), with no significant time × group effect (*p* > 0.05; *d* = 0.412).

## Discussion

To the best of our knowledge, this study was the first to examine the influence of collagen peptide supplementation following a 12 week concurrent training on endurance performance, cardiometabolic parameters, body composition and muscle endurance in recreationally active women. The major findings were that supplementation with SCP further improved the positive effects of CT regarding time trial performance, HR at the IAT and the increase in FFM in the subjects investigated.

In the present study, the SCP group showed an improved endurance performance in the time trial which could be explained by an improved aerobic metabolism. Moreover, although not statistically different, a more pronounced improvement in velocity at both the LT and the IAT in favor of the SCP group could be observed. The IAT represents a reliable parameter to assess endurance performance capacity (64). Since, Achten et al. (52) have demonstrated that the onset of plasma lactate accumulation occurs at the same exercise intensity as for maximal fat oxidation both thresholds indicate a superior rightward shift fuel utilization toward fats. In addition, the SCP group showed a considerably lower heart rate at the aerobic and anaerobic threshold indicating an improved cardio-circulatory adaptation.

The influence of protein intake following CT on cardiometabolic parameters is largely unknown. Although it has been previously shown by one study that protein supplementation further stimulates fat metabolism during exercise thereby improving endurance performance, the evidence is rather limited (65). Early research data indicated that a 2-week co-ingestion of proteins and carbohydrates significantly elevated PGC-1α expression following an exercise bout compared to carbohydrate feeding alone (66). PGC-1α signaling is involved in mitochondrial biogenesis and improved fat metabolism (67). Evidence from an acute study showed that PGC1α expression was twofold higher with CT compared to a single-mode of endurance training (68). Moreover, a review indicates an up-regulation in AMPK-PGC1α in response to RT and ET, especially when protein is supplemented (69).

Another downstream signaling of PGC-1α involved in fatty acid metabolism is peroxisome proliferator-activated receptor (PPAR). PPARs are a nuclear receptors family consisting of the transcription factors PPARα, PPARδ, and PPARγ and regulate fat metabolism in the body. Acute and chronic exercise leads to an increase of PPARα in muscle cells and PPARγ in liver cells which has been associated with an improved lipid metabolism (70). It has been shown that feeding collagen peptides to mice over a period of 8 weeks resulted in a significant increase in PPARα expression and fatty acid metabolism compared to a control group (39). Similar findings were observed in a 10-week intervention in which PPAR signaling pathway and fatty acid metabolism significantly increased. The authors concluded that collagen peptide ingestion upregulates PPARα expression which leads to an increased lipogenesis, fatty acid beta-oxidation and fatty acid transport (38).Therefore, it might be speculated that the AMP-activated protein kinase (AMPK)-PGC-1α signaling cascades including PPARs, may explain a potential mechanism of increased fat metabolism by supplementing SCP in the present investigation.

In general, the key metabolic adaptations to prolonged endurance training shifts fuel utilization toward fat metabolism (8–11) which is accompanied by an increased mitochondrial content and thus an enhanced aerobic capacity (7). For the SCP used in the current trial the positive impact on mitochondrial content was investigated in a preclinical trial with a rat model. A statistically significantly increased mitochondrial density (in fine-needle biopsies of the Quadriceps femoris) of ca. 57% were determined by transmission electron microscopy after 4 weeks of an oral SCP dosage which was equivalent to a daily intake of 15 g in humans. In contrast, in rats fed with the tap water control no changes in muscle mitochondria content was determined (71). However, the exact mechanisms on how SCP might directly stimulate human fat metabolism remain unclear and need to be further investigated.

It has to be noted that the augmentation in endurance running performance is not solely attributed to aerobic and anaerobic capacity but also to neuromuscular motor competence and running economy (72–74). A systematic review from controlled clinical trials in men and women has shown that also resistance training improved time trial performance, running economy and running velocities in endurance athletes (74). Interestingly, although long-term investigations are scarce, it is generally believed that protein supplementation may further improve endurance performance following a CT (15, 75).

In addition, a previous study showed that CT training led to improved muscle strength, running economy and running velocity with no significant effects on VO2 kinetics pattern in male runners (76). Most of the CT studies (77) use a hypertrophy-oriented full body training with 70–85% of 1RM and 8–12 repetition according to the ACSM guidelines (5). Although body weight resistance training can be an efficient strategy to improve metabolic health, it may be inferior to creating absolute strength compared to commonly used strength trainings regimes (78). However, this trainings method was used to increase maximal aerobic capacity and decreasing body fat (61). Interestingly, a current meta-analysis revealed that hypertrophy adaptations between low- vs. high-load resistance training can be equally achieved across a spectrum of loading ranges (79), which might also be responsible for improvements in running economy and velocity due to an improved muscle strength and endurance in the current investigation. Moreover, body weight resistance training was also used due to its practical application, since the use of equipment or a facility is not required (61).

The current trial is in line with a previous investigation, showing that running CT enhances fat free mass and muscle strength (80). In a recent meta-analysis, Wilson et al. (40) demonstrated superior increases in muscle hypertrophy as well as higher reductions in fat mass following CT compared to endurance training alone. The increase in FFM is attributable to both, a rise in skeletal muscle mass and total body water on a clinically relevant level, as indicated by the calculated effect sizes. Bosy-Westphal et al. (81) have confirmed that the phase-sensitive 8-electrode medical bioelectrical impedance device, which was used in the present investigation, is a highly accurate device for quantifying whole-body skeletal muscle mass (81). Although the influence of different training regimes on FM and FFM has been extensively explored, to the best of our knowledge, no investigation has studied the effects of long-term CT on body water distribution. Previous investigations indicate that maintaining or increasing ICW with a simultaneously elevated FFM and TBW are related to performance, irrespective of sex and type of sports (82–84). However, due to the different populations, investigation or intervention periods and measurement methodologies these findings should be taken with caution when extrapolating it to our results.

It was not the intention of the present study to generate evidence for an increase in muscle mass as this was not within the scope and the primary outcome variables of the investigation. All necessary precautions were taken to assure the outmost pre-analytical and analytical precision before and during the BIA analysis. However, further experiments are needed to investigate the interrelation between changes in FFM, SMM and TBW following SCP supplementation and exercise interventions.

In addition, as a trend, superior repetitions in the muscular endurance test were observed in the TG (+36.3%) compared to the CG (+21.6%). With regard to the interference effect, these results are in line with an earlier meta-analysis regarding simultaneous strength and running endurance training (40). There is an ongoing debate whether the anabolic process can be further enhanced by protein supplementation following a CT (15, 75, 85). Although the data situation is not clear, the interference effect is generally explained by a changed balance between muscle protein synthesis and muscle protein degradation, which are induced by antagonistic signaling pathways of anabolic and endurance stimuli on a molecular level (86).

Recently, experimental results reported that protein administration after a single bout of CT increases myofibrillar protein synthesis and attenuate markers of muscle catabolism in men (87). On the other hand, Ormsbee et al. (88) showed no additional increases in muscle mass and strength in the group with protein supplementation after 6-month of CT in sedentary women. Although there is currently limited knowledge on the signaling effects of collagen peptides on muscle anabolism, the activation of mTOR signaling pathways through collagen peptide intake has been shown *in vitro* and in a clinical trial (22, 34). A previous investigation in women confirmed the present data by showing a significantly higher increase in FFM following SCP supplementation in combination with resistance training compared to placebo (21). It is important to note, however, that investigations by Ormsbee et al. (88) and Shamim et al. (85) differ in amount, quality and timing of protein. In addition, the initially loading protocols, the recorded parameters and the protocols used to verify endurance and strength performances differ from each other. Therefore, a direct comparison of the effects on these parameters is not possible.

Interestingly, a recent review and meta-analysis reports that an enhanced biochemical properties of the muscle-tendon system lead to improved running economy (72). This might be explained by an improved energy storage capacity and force potential of the muscle with increased tendon stiffness (89, 90). Since human tendons comprise 60–85% of collagen (91), the supplementation of collagen peptides might represent a promising strategy for improving tendo-muscular properties. First evidence from a controlled double-blind trial in men and women with chronic tendinopathy has shown that the administration of collagen peptides in combination with calf muscle resistance training was associated with significantly improved function of the Achilles tendon (92). An improved running economy might also contribute to the marked reduction in heart rate at both, the aerobic as well as the anaerobic threshold. Therefore, although it is still speculative, the differences in running velocity between the groups could also be explained by an improved structural and mechanical property of the muscle-tendon system in the TG group.

With respect to the interpretation of our results, there are some limitations in the present investigation that should be mentioned. Firstly, the study examined cardiometabolic parameter by using heart rate and blood lactate at the LT and IAT as surrogate markers and not muscle biopsies (93) and tracer approaches (94) as gold standards. However, it has been shown that measuring these surrogate markers is an effective way to indirectly assess changes in aerobic and anaerobic metabolic processes (51, 52).

Secondly, there is an ongoing discussion about gender-specific differences in the sensitivity of BIA measurements regarding exercise-training induced changes in body composition of study participants (95, 96). Nevertheless, it has been demonstrated in various experimental settings, that the multifrequency bioelectrical impedance assessment used in this study is a valid and reproducible method to estimate body composition in healthy adults (57, 81, 97).

It must be also emphasized that these results are valid for the investigated specific collagen peptides only and cannot be generalized to other collagen peptides.

## Conclusion

In conclusion, our results indicated that supplementation of SCP in combination with CT resulted in a statistically significant increase in the time-trial performance compared to the control group. This might be a potential consequence of improved cardiometabolic parameters, mainly aerobic metabolism, as measured in the incremental treadmill test. Moreover, HR at the IAT was improved after SCP intake, indicating an improved biomechanical motor competence. Both, the metabolic and structural adaptations could have led to an enhanced running velocity and improved running economy. In this context, the additional intake of SCP after CT appears to be beneficial to further improve the effect of endurance training on fat free mass and muscular endurance.

## Data Availability Statement

The datasets generated for this study are available on request to the corresponding author.

## Ethics Statement

The studies involving human participants were reviewed and approved by the ethics committee of the University of Freiburg (43/18) and was in accordance with the latest revision of the Declaration of Helsinki. The participants provided their written informed consent to participate in this study.

## Author Contributions

PJ, JK, CC, AG, and DK designed the study. PJ and DK were responsible for data acquisition. Analysis was performed by PJ, JK, and DK. All authors read and approved the final version of the manuscript. All authors contributed to the article and approved the submitted version.

## Conflict of Interest

The authors declare that the research was conducted in the absence of any commercial or financial relationships that could be constructed as a potential conflict of interest.
